# Ourselves

**Published:** 1855-12

**Authors:** 


### OURSELVES.

Our subscribers are requested to notice, that this number completes the volume for which they have paid. We have not a delinquent patron. Those who wish to take the journal for another year, must do two things:

First, write us a note to that effect.

Second, enclose one dollar in such note.

A *third* thing, we merely request to be done, to wit, that each one who feels interest enough in this publication to renew his subscription, would induce some of their friends to subscribe with them. We know very well that no family can read our pages without deriving from them much valuable information, for which a dollar is no kind of return, and we are just as confident that such a publication is needed in every family in the United States; its object and the tendency of its teaching being TO MAINTAIN HEALTH, AND TO RECOVER IT WITHOUT THE USE OF MEDICINAL MEANS, THAT BY PROPER ATTENTION TO THE HABITS OF LIFE, THIS WILL ANSWER IN MULTITUDES OF INSTANCES, BUT WHEN NOT SUFFICIENT, TO APPLY PROMPTLY TO THE REGULARLY EDUCATED PHYSICIAN, WITH ENTIRE SUBMISSION TO HIS DIRECTION.

No man or woman of cultivated intelligence can feel otherwise than that the practical prevalence of such sentiments would largely result in the health, prosperity, and happiness of any community, and to such cultivated minds we look for efficient co-operation in widely extending the circulation of this Journal. To make it remunerative directly, pecuniarily, to an extent which would give us a liberal compensation for our personal labor in conducting it, would require that each present subscriber should send in a dozen names, with as many dollars, besides his own.

If we have any other request to make, it is this, that each present subscriber, order the *Journal* for his minister; it is such a publication as every clergyman ought to read, for it gives to them every month available, appropriate, practical knowledge, which they cannot get anywhere else. We know that very many clergymen would take it, if they could feel their way clear to appropriate a dollar in that direction, but as a very general rule, they are so poorly paid, and it is a disgrace to the age that such should be the case, that even a dollar's expenditure must be preceded by the mature inquiry, *is it an indispensable necessity?* We have not one clerical subscriber in all New York city, and a special canvasser among them, reported, that a not unfrequent answer was, "I do not feel able to spare a dollar for that purpose." If that be the case in a community where the clergy are so well cared for as in New York city, how much greater are the straits of the country clergy, just as able, just as good, whose yearly compensation does not average five hundred dollars, all told, out of which a whole family is to be supported. No wonder we see so many of the best spirits among them dying before their time, or prematurely giving out under the weight of that distressing inquiry as they look upon their wives and little ones, hundreds of times, in a year, "*How shall we get along any farther?*" This is not a fiction, it is a fact of which we are personally cognisant, having from previous as well as present associations and circumstances, a more enlarged opportunity of knowing these things, than perhaps, any one individual in the United States.

For want of knowledge, many a clergyman has slidden into ill-health and passed prematurely away, who else might have lived to widely useful purposes, and many more are invalided, who ought to have preached a quarter of a century longer. The main, the first object of this journal, next to my own pecuniary benefit, was to meet these very difficulties, believing, as I do, that the best care we can take of the ministry, pecuniarily, and otherwise, is not a whit more than they are entitled to, by reason of the fact, that it is by their teachings and influence that the present age is what it is, in all that is noble, elevated, refined, and that without the influence of the Bible principles, which they spend their lives in inculcating, there would not be a government on earth that was not an anarchy, and no subjects that were not Atheistic in principle; and in practice, all that was selfish and savage.

These things being so, the robust health of the clergy is essential to their widest success, hence the appropriateness of our appeal to each individual subscriber, to place a *Journal* like this in the hands of some one clergyman, who might learn thereby how to maintain that health which is essential to his being a fully efficient laborer in his Master's vineyard.

